# Environmental filtering and community delineation in the streambed ecotone

**DOI:** 10.1038/s41598-018-34206-z

**Published:** 2018-10-26

**Authors:** Ignacio Peralta-Maraver, Jason Galloway, Malte Posselt, Shai Arnon, Julia Reiss, Jörg Lewandowski, Anne L. Robertson

**Affiliations:** 1 Department of Life Sciences, Roehampton University, London, UK; 2 Leibniz-Institute of Freshwater Ecology and Inland Fisheries, Department Ecohydrology, Berlin, Germany; 3 Department of Environmental Science and Analytical Chemistry, Stockholm University, Stockholm, Sweden; 4 Zuckerberg Institute for Water Research, The Jacob Blaustein Institutes for Desert Research, Ben-Gurion University of the Negev, Midreshet Ben-Gurion, Israel; 5 Geography Department, Humboldt University, Berlin, Germany

**Keywords:** Freshwater ecology, Community ecology

## Abstract

A current controversy in ecology is whether biological communities are discrete biological entities or simply study units created for convenience; a debate that becomes even more heated when delimiting communities along ecotones. Here, we report an interdisciplinary study designed to address the interplay between environmental drivers and community ecology in a typical ecotone ecosystem: the streambed. Environmental filtering at a micro-scale determined how diversity, productivity and composition of the whole streambed assemblage varied with depth and with the direction of vertical water exchange. Biomass and production decreased with increasing depth, and were lower under upwelling than downwelling conditions. However, the rate at which biomass and production decreased with increasing depth differed significantly for different taxonomic groups. Using quantitative biocenosis analysis, we also showed that benthic and hyporheic zone assemblages (assemblages in close juxtaposition) could be clearly distinguished as discrete communities with individual integrity. Vertical hydrodynamic conditions also influenced the demarcation between both communities; the benthic community reached greater depths in downwelling than in upwelling zones.

## Introduction

Delineating communities has a long history reaching back to the writings of Theophrastus in the 4^th^ century BC^1^. Even today the integrity of communities as real biological entities is disputed^2–4^. This controversy stems from a common problem within ecology, which is that the community concept is a term frequently used but often only vaguely defined^4^. Thus it is important to distinguish communities [sets of biological populations inhabiting a certain biotope differing sufficiently (qualitatively and quantitatively) from other sets to be considered an ecological entity] from an assemblage [groups of organisms that are found together but where there is insufficient evidence to state that they form distinct communities]^4^. Delineating communities might be hard in transitional ecosystems and at a local scale^5^, for example within a vertical gradient in streambed sediments (the surface–groundwater ecotone).

Traditionally the sediments of streams and rivers and their assemblages have been divided into two compartments according to their depth in the stream bed: the benthic zone (BZ), in direct contact with surface water and exposed to light and scouring forces of water, and the hyporheic zone (HZ), defined by shallow subsurface water pathways through river beds and banks beginning and ending at the river^6^. This latter zone is also a biogeochemically active interface with a significant role in the functioning of aquatic ecosystems and in the retention and attenuation of nutrients and contaminants^7^. The line of demarcation between benthic (*benthos*) and hyporheic (*hyporheos*) assemblages can be recognised as the boundary between the benthic and hyporheic zones (the biological definition of the hyporheic zone)^8^. However, distinguishing between the benthos and the hyporheos can be challenging due to the dynamic and ecotonal nature of the hyporheic zone^9^ and the question remains as to whether these two assemblages are real biological entities or merely units created by freshwater ecologists for convenience.

Depth below the surface and the direction (and magnitude) of the surface-groundwater exchange are acknowledged to be primary drivers of species distribution, assemblage structure and ecology in the streambed^10–17^. At greater depths in the streambed sediment colmation occurs leading to reduced pore space and a reduction in oxygen availability, conditions that limit the vertical distribution of organisms with higher metabolic rates and larger sizes^18–21^. Thus, the ability of streambed organisms to colonise subsurface sediments depends on the biological traits that they possess^20–22^ and suggest that sediments will contain assemblages with relatively large invertebrates at the surface and that, with increasing depth, these will be replaced by a suite of numerous but small-bodied organisms^23,24^. Water mixing in the HZ can lead to complex temporal and spatial flow patterns^25^ in which downwelling (DW) and upwelling (UW) conditions may occur alternately^26^ and the vertical extent of the HZ can be variable^9^. The aforementioned hydrodynamic patterns are reflected also in the biogeochemical conditions in the HZ. Typically, water downwelling from the surface contains higher levels of easily degradable organic matter and oxygen^14,15,27^. Therefore, abundance and diversity of streambed assemblages might decline with increasing depth and is higher in DW than in UW zones^13,16,28^. The selective pressures of the depth-dependent hydrodynamic and biogeochemical conditions can be considered micro-scale filters in streams and rivers (*sensu*)^29^ through which species must pass to constitute part of a given community^29^.

Most studies relating streambed assemblages with these environmental filters (depth and vertical hydrodynamics) have focused on single size groups: eumetazoan invertebrates (macroinvertebrates and meiofauna: multicellular organisms whose body size is greater 0.45 μm)^13,28,30^ or Protozoa (eukaryotic single cell organisms)^16^. Given that organisms differ in their ability to colonize the streambed sediments depending on their metabolic capabilities and body-size, we would expect a significant interaction between depth and taxonomic group (flagellates, ciliates, and multicellular invertebrates). Previous predictive regression models that explain the vertical gradient of biomass and secondary productivity in the streambed as responses of the depth gradient focus exclusively on large size organisms (mainly macroinvertebrates and a few meiofauna groups) and do not consider hydrodynamic conditions. Thus, they explain only a small part of the observed variation highlighting the necessity of including more predictive variables and interactions^17^.

To tease apart the nature and hierarchy of variables driving the structure and functioning of streambed systems, and to determine whether the benthos and hyporheos are indeed real biological entities, we took an interdisciplinary approach. We combined techniques from hydrology and community ecology to determine flow in streambed sediments and to characterise the resident assemblages at the same spatial and temporal scales. We modelled the effect of vertical water flux and streambed depth on the diversity, productivity and structure of streambed assemblages in a lowland river at a high spatial resolution. We included eumetazoan invertebrates and two size-groups of protozoa (ciliates and flagellates) and thus, for the first time, our analysis spanned more than ten orders of magnitude in terms of body size. Comparing density across such a range of body sizes is problematic and so we focussed on comparisons of biomass, productivity and diversity.

Our overall aims were to demonstrate that the benthic zone and hyporheic zone are indeed different environments containing discrete communities that can be clearly delimited and that environmental filtering, resulting from the interplay of vertical hydrodynamics and depth, rules the vertical gradient of biomass, production and diversity in streambed assemblages. We hypothesise that (1) diversity, biomass and secondary productivity (responses) will decrease following the depth gradient, however the reduction in biomass and secondary productivity with increasing depth will depend on the taxonomic group. Accordingly, differential abilities to colonize the streambed sediments will result in an important interaction term of our predictive models for biomass and secondary productivity. (2) Biomass, productivity and diversity are expected to be significantly higher under DW flow conditions, where there is higher dissolved organic carbon and oxygen, than under UW flow conditions. Thus, direction of vertical flow will be also an important predictor variable in our models. (3) With increasing depth, the benthic community will be replaced by a significantly different hyporheic community enabling the boundary between both communities to be delineated. (4) Vertical hydrodynamic conditions (DW vs. UW) will determine the depth at which this boundary occurs; it will be deeper under DW conditions as a result of the downward influence of surface stream water.

## Methods

### Study site

**Figure 1:**
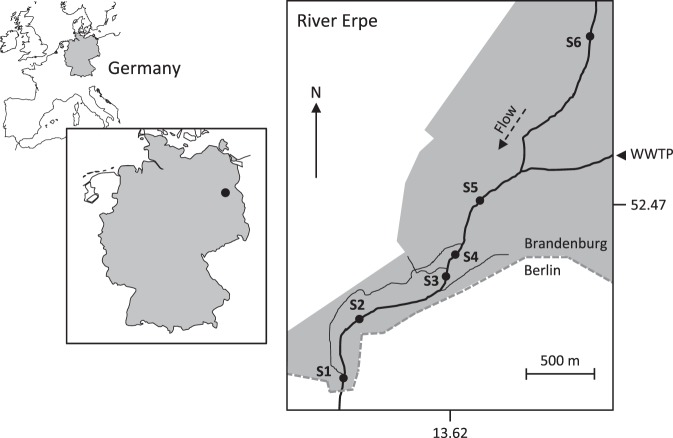
The study site at the River Erpe with sampling sites (S1–S6). Effluent input from the municipal wastewater treatment plant (WWTP) Münchehofe is also shown. Grey shading in the right panel represents the protected area surrounding studied site. Figure 1 was generated using the *map* function from *maps* R-package version 3.2.0^74^.

**Figure 2:**
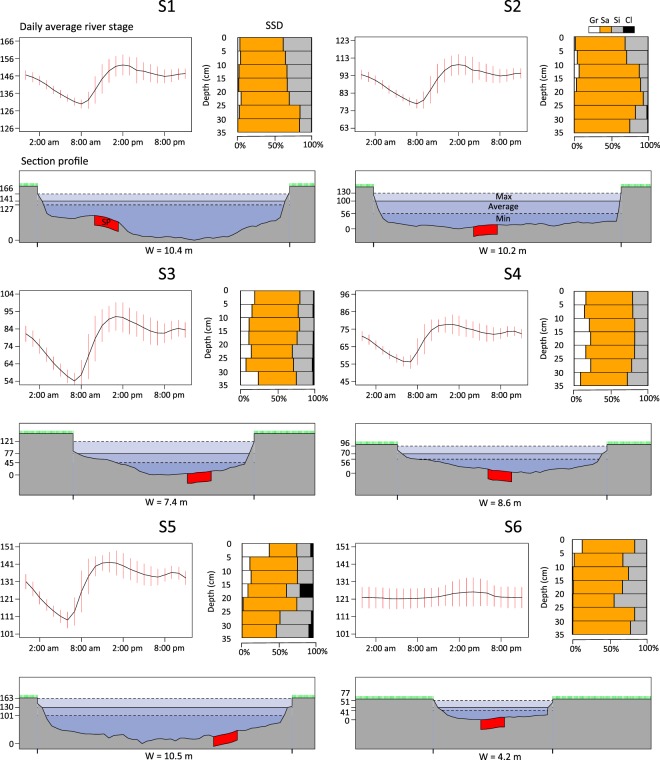
Hydrological and geomorphological features of each sampling site (S1–S6). Each panel shows: (top-left) the daily average river stage (cm) of the period 16/05/2016 to 16/06/2016 with the mean value (black line) and the standard deviation of every hour (24 vertical red bars); (top-right) the proportion sediment size distribution (SSD: Gr = gravel, Sa = sand, Si = silt, Cl = Clay) for each 5 cm layer (between 0 and 35 cm); (bottom) the river cross section with average, maximum and minimum water stage during the above-mentioned study period. The location of sampled volume of sediments (SP) is also shown in red. W stands for width of the river.

The study was conducted on the lowland river Erpe, Northeast Germany (Fig. 1), between 16^th^ May and 16^th^ June 2016. The catchment area is affected by agriculture activities and the river receives daily treated wastewater releases from the Münchehofe wastewater treatment plant (WWTP). As a result, streambed sediments are rich in organic carbon and nutrients^31^. Six sites were studied along a 3.5 km river stretch including one site upstream of the WWTP and five sites downstream (Fig. 1). Study sites showed similar streambed morphology in which local-scale conditions were not affected by elements such as dunes, riffles or bars. These locations were selected as potential UW or DW sites based on preliminary analysis of the hydraulic gradient between the river and groundwater table. At each site, a sediment portion of 1 m^2^ area and 35 cm depth was selected in the streambed as a sampling point (Fig. 2). Following Lewandowski and Nützmann^32^, two sediment cores (6 cm diameter) per sampling location were taken with a modified Kajak corer (Fa. Uwitec). Cores were sliced in discrete 5-cm layers down to 35 cm and placed in plastic bags. Grain size distribution (GSD) was determined per layer after drying at 105 °C for 24 h. Percentage of GSD was classified by sequential sieving according to^33^. Additionally, water level data loggers (CTD-Diver, Westbay Instruments, Burnaby, Canada) were deployed in the water column at the west margin of every sampling point (Supplementary Material: Fig. S1) providing water pressure data every 10 min (pressure accuracy ± 0.5 cm H_2_O). These data were used to provide elevation in cm relative to sea-level (m a. s. l. = meter above sea level). These measurements were combined with cross-section profiling by measuring water depth every 10 cm along transects across the river (Fig. 2).

### Vertical hydrodynamics

Vertical hydrodynamic conditions at different sites were characterised by coupling the location of the thermal extinction depth, measurement of the vertical streambed fluxes, and indirect analysis of redox conditions. For that purpose, lances with 8 temperature sensors (at 2.5, −2.5, −12.5, −17.5, −22.5, −27.5, −37.5 and −57.5 cm depth; resolution 0.04 °C; measurement frequency 10 min; UIT, Dresden, Germany) were installed vertically in the sediment (Supplementary Material: Fig. S1). Thermal extinction depth (or specific penetration depth of a periodic surface temperature signal) was determined as the depth at which daily temperature variation is undetectable (amplitude of daily temperature variation becomes undetectable).

Vertical streambed fluxes at each site were calculated through time series analysis of streambed thermal depth profiles during the whole study period^34–36^. The measured thermal time series were analysed with numerical one-dimensional advection-diffusion equations implemented in VFLUX 2 (MATLAB toolbox)^36–38^. This software calculates the one-dimensional vertical flux through saturated porous media based on Hatch *et al*.^34^ and Keery *et al*.^35^. Input parameters used here to compute the one-dimensional advection-diffusion equations are available as Supplementary Material: Appendix 1.

Finally, redox conditions in pore water through depth were characterised by profiling the concentration of aqueous ferrous iron (Fe^2+^, highly reactive with oxygen) using one dialysis sampler (peeper) per study site. Peepers had seven chambers with a centre-to-centre separation of 5 cm (35 cm total length, vertical resolution of 5 cm). The capacity of each chamber was 20 ml. Detailed explanations of peeper preparation and set-up can be found in Hesslein^39^. Peepers were inserted vertically into the sediment to a depth of 30 cm with one chamber above the sediment and allowed to equilibrate with pore water for 21 days (Supplementary Material: Fig. S1). After this period, they were removed from the sediment checking integrity of the dialysis membranes. One broken membrane was detected at site 5 in 10 cm depth and excluded from the analysis. Water from the rest of the chambers was collected and samples were put on ice until analysis, which took place within 12 hours of sample collection. Finally, ferrous iron (Fe^2+^) concentration was measured photometrically using a segmented flow analyser (Skalar analytical B. V., EN ISO 11732 – Water quality).

### Streambed community

#### Sampling

The community of invertebrates and protists inhabiting the sediments was sampled using a modified Kajak corer. This corer has been shown to be very reliable and to provide representative samples of the streambed assemblages in sandy and silty habitats^30,40–42^. Three cores were taken per sampling point every seven days over 4 weeks. On each sampling date, the locations of the sampled cores were slightly altered to reduce disruptive effects on community assemblage (Supplementary Material: Fig. S1). Discrete 5-cm layers were sliced down to 35 cm, the lower limit of the community distribution. The extraction system of the corers avoided cross-contamination during the slicing procedure. Equivalent layers from the three corers were then pooled (to maximise the representativeness of our samples) and well-mixed in individual plastic bags (giving 7 samples). From these plastic bags, subsamples for Protozoa processing (flagellates and ciliates) were transferred to 10 ml Sterilin plastic bottles and cooled until analysis. The remaining bag sample was then fixed with formalin at 4% containing Bengal rose stain.

#### Identification and quantification of organisms

In the laboratory, ciliates and flagellates were identified and counted alive within 24 h of sampling. Sub-samples were taken from the Sterilin bottles and processed under an Olympus BX50 microscope. Ciliate sub-samples were counted and identified using a Sedgewick Rafter counting cell chamber (1 ml volume; Pyser-SGI limited, Edenbridge, United Kingdom), while flagellates were counted using a Neubauer cell counting chamber. Ciliates were identified to sub-class using identification keys^43^, flagellates were treated as a single group. The remainder of the sample was rinsed through a 40-µm sieve to remove sediment and preserved again in formalin 4% containing Bengal-rose stain. Individuals were subsequently extracted under a Nikon SMZ-U stereomicroscope (30x), identified to the maximum possible resolution (Supplementary Material: Appendix 2) using identification keys^44,45^ and counted. The length and width of all counted organisms (Protozoa and invertebrates) were measured to the nearest micrometre.

#### Biomass, secondary production and diversity

Body dimensions of all counted organisms were converted to biovolume as described by Reiss and Schmid-Araya^46^. Then frequency of biovolume measurements was studied to verify the reliability of arranging eumetazoan invertebrates, ciliates and flagellates as different size-groups. Following Putt & Stoecker^47^, protozoa individual biovolume was directly transformed to carbon content assuming 0.14 pg C/µm^3^, while for invertebrates it was first converted into fresh mass implementing published gravity values^48^. Although this approach is widely used in the literature (i.e.^49,50^), caution is needed because some taxa may give site specific responses. Invertebrate individual carbon content was then calculated by using dry/wet mass ratio of 0.25 and dry mass/carbon content of 0.4^48^. Biomass (mg C/L) of all identified taxa was calculated for each sampling date and depth-layer by multiplying carbon content with individual density (ind/L).

To be consistent, the same non-cohort method was used to calculate secondary production of all taxonomic groups. Although this method is a suitable approximation to deal with the complex life histories of our assemblages and is widely used^51^, it does not account for losses in production from factors such as migration, disease and predation^51^. Total secondary production (*P*_*t*_; mg C/L month) of identified taxa was calculated after Reiss and Schmid-Araya^46^ as the sum of interval production (*P*_*i*_) between sampling dates (n = 3). Interval production was obtained as the product of mean biomass within the interval $(\hat{B})$, turnover rate of biomass per day (*r*) and interval duration in days (Δ*t*, 7 days):

1 $${P}_{i}=\hat{B}\times r\times {\rm{\Delta }}t$$

2 $${P}_{t}=\sum _{i=1}^{n}({P}_{i})$$

In the case of ciliates, flagellates, and permanent meiofauna (i.e. the non-insects), turnover rate of biomass was defined as the intrinsic rate of population increase. This rate was obtained by applying the allometric scaling relationship proposed by Reiss and Schmid-Araya^46^, which relates turnover rate of biomass with body mass of taxonomic groups. Subsequently, the critically temperature dependent intercept in equation 1 was temperature-corrected per interval and along the depth gradient (using temperature data collected at the same time and scale with the thermal lances) by applying equations given by Gillooly *et al*.^52^. Turnover rate of biomass of meiofauna and macroinvertebrates was defined as daily growth rate (instantaneous growth method)^51,53^. Daily growth rate values were obtained using published equations for different taxonomic groups^28,54^. Similarly, these values were also temperature-corrected per interval and along the depth gradient.

Finally, the diversity gradient along the depth per site was measured for every sampling date using the Shannon-Wiener´s diversity index (*H*′):

3 $${\rm{H}}^{\prime} =-\,\sum _{{\rm{i}}=1}^{{\rm{S}}}\,P{r}_{{\rm{i}}}\,\mathrm{ln}(P{r}_{{\rm{i}}})$$

where S is the number of taxa in the community (here the assemblage at each depth-layer) and *Pr*_i_ the proportion of individuals in the community that belong to taxa i^55^. This index is a useful method for following variability in relative density in a large number of taxa over time^56,57^.

### Statistical analysis

#### Biomass, secondary production and diversity

Two linear mixed effect models (LMMs) were applied to test the effect of vertical hydrodynamic conditions (factor with two levels: UW and DW), depth (continuous covariant) and taxonomic group (factor with three levels: eumetazoan invertebrates, ciliates and flagellates) on the responses biomass and production respectively. Additionally, the effect of vertical hydrodynamics and depth on Shannon-Wiener diversity was modelled using a Linear Mixed Model with Poisson distribution and natural logarithmic link function (Poisson-GLMM). In order to solve heterogeneity in the residuals, biomass and production variables were log_10_ transformed. Non-controlled variables (i.e. effect of WWTP input, altered position of samples from one date to another) and temporal replication produced an intra-class correlation effect of the responses with the study site (a residual pattern with study site was observed during data exploration). Therefore, study site (*Site*) was incorporated in our models as a random factor (random intercept) in order to cope with this non-independence. Subsequently, a Widely Applicable Information Criterion (WAIC) was used to find the optimal models by combining all main terms and potential first–order interactions. Model validation was applied to verify the underlying assumptions^58^, checking also the absence of overdispersion in the Poisson-GLMM (Person residuals/freedom degree = 0.04). Previous models were fitted using functions *lmer* and *glmer* of the R package lme4^59,60^. Finally, 5000 values from the posterior joint distribution of the model parameters were simulated with the function *sim* of the R package arm^61^. This function uses an analytical direct-simulation method with uninformative priors^62^. Obtained means of the simulated values from the joint posterior distribution of model parameters were used as estimates, and the 2.5% and 97.5% quantiles as lower and upper limits of 95% credible intervals. Finally, the conditional-R^2^ was calculated to assess model fit using the function *rsquaredGLMM* of the R package MuMIn^63^.

#### Delineation of communities

assemblage structure was assessed weekly at 5 cm depth intervals (7 depths in total) through the sediment both in DW and UW zones in order to delineate the border between benthos and hyporheos. The Bray-Curtis similarity index was applied; this is a quantitative index that takes composition and proportional density of the organisms inhabiting each layer into account. An analysis of similarities (ANOSIM) was performed to test at which depth differences between upper layer and the subjacent sediment become statistically significant. Accordingly, significance of ANOSIM statistic (*R*) was compared with its null distribution by permuting group membership 1000 times. Finally, assemblages at different depths and under different vertical hydrodynamic conditions were compared and clustered depending on their averaged Bray-Curtis similarity value. In this manner, similarity of benthos and hyporheos between sites was checked. Bray-Curtis index and ANOSIM analysis were performed using *vegdist*, *anosim* and *hclust* functions of the R-package vegan^64^.

## Results

### Study-site characterisation

**Figure 3:**
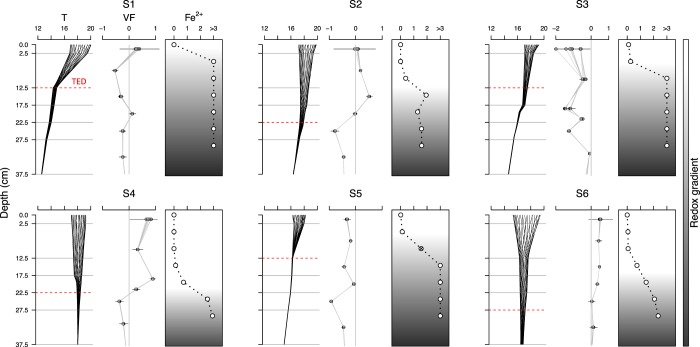
Daily amplitude of temperatures (T, °C), vertical flux profiles (VF, m/day) and ferrous iron concentration (Fe^2+^, mg/L) at each sampling site. Temperature profiles consist of 24 lines for each hour of a diurnal cycle and are averaged based on 4 weeks of data (16/05/2016 to 16/06/2016). Thermal extinction depth (TED) is marked as a red dotted-line between the two temperature sensors in which the amplitude of daily temperature variation becomes undetectable. VF: The mean vertical flux between two neighbouring temperature sensors calculated with the 1-D numerical model VFLUX (Matlab) using the Hatch amplitude method^29^. Dots represent mean temperature values for every 2 h during the day (12 dots per depth), while horizontal lines represent the standard deviations. Positive VF mean values indicate downward flux, negative values indicate an upward flux. Fe^2+^: Vertical profile of ferrous iron concentrations based on one peeper deployment per sampling site (indirect measurement of the redox conditions).

All study sites showed a relatively similar GSD through the depth profile, aside from marginally higher proportions of interstitial clay at sites 5 and 3, and higher proportions of gravels at sites 4 and 5 (Fig. 2). The effect of the WWTP release was evident on the river stage of downstream sites (1–5), which reached minima during the early morning and then rose sharply until noon remaining high until the evening (Fig. 2). Despite the daily fluctuation in the surface water, it was possible to characterise sites 1, 3 and 5 as UW zones and sites 2, 4 and 6 as DW zones (Fig. 3). Therefore, we could accept that daily fluctuations in the surface water level did not affect substantially the general physicochemical and vertical hydrodynamic conditions at each study site during the study period. The rate at which the daily amplitude of temperatures decreased was greater in sites 1, 3 and 5 than in sites 2, 4 and 6 (Fig. 3). Therefore thermal extinction depth, and consequently surface water influence, did not penetrate so deep at sites 1, 3 and 5. These results agree with the obtained streambed vertical-flux values and redox conditions. Streambed vertical-flux values after VFLUX2 routines were similar using both the Hatch and the Kerry Amplitude method, thus, only the results from Hatch amplitude method are reported here. At sites 1, 3 and 5 the upward flux of water between temperature sensors (negative mean values, Fig. 3) was dominant throughout the study period. In contrast, at sites 2, 4 and 6 downward flux of water into deeper layers (positive mean values, Fig. 3) was observed. Finally, Fe^2+^ concentration in pore water (as an indirect measure of the redox zonation) increased much faster with increasing depth in site 1, 3 and 5, while its concentration remained lower in the upper layers of site 2, 4, and 6 (Fig. 3). Mean and standard deviation of temperature, vertical flux and Fe^2+^ concentration throughout depth are available as Supplementary Data: Datasets S1–S3.

### Biomass, secondary production, and diversity

**Figure 4:**
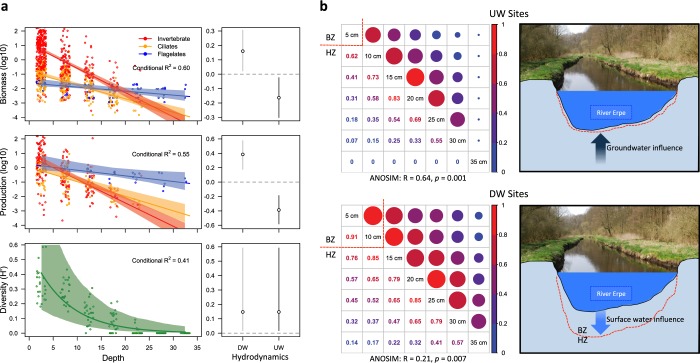
(**a**) Multiple linear regression models for biomass, production and Shannon-Wiener diversity. Coloured shaded area on the regression line and vertical bars represent the 95% credible intervals. (**b**) On the left, Bray-Curtis similarity matrix of the assemblage structure between depth layers in upwelling sites (UW) and downwelling sites (DW). Each matrix contains the numeric Bray-Curtis similarity value that resulted from comparing assemblage structure (composition and abundance) between sections. Similarity values range from maximum similarity (1.00) to maximum dissimilarity (0.00). A colour/size code is given to facilitate interpretation where big red circles represent maximum similarity, while small and blue circles represent maximum dissimilarity. The red dotted line marks the depth at which differences with the upper layers were significant for the ANOSIM analysis. This is represented schematically in the panels on the far right. Photographs in panel b were taken by Jörg Lewandowski (co-author of this study).

A total of 3874 eumetazoa invertebrates, 2165 ciliates, and 420 flagellates were collected and identified to measure diversity, biomass and secondary production (a taxa list with the community composition through depth and at different vertical hydrodynamic conditions is available as Supplementary Material: Appendix 2). The frequency of body sizes in the collected organisms clearly discriminates between the three studied groups (Supplementary Material: Appendix 3). However, measurements of diversity, biomass and secondary production were highly variable depending on taxonomic group, depth and vertical hydrodynamic conditions (Supplementary Material: Appendix 4; Analysed spread sheets are available as Supplementary Data: Datasets S4 and S5). After WAIC routines, biomass and production models included vertical hydrodynamic conditions as a single factor, depth as a continuous covariate and the interaction between depth and taxonomic group. In the case of the diversity model, depth and vertical hydrodynamic conditions were kept as effective parameters, while the interaction was not included. Fitted statistical models had a relatively high explanatory capability (Conditional R^2^ > 0.4 in all cases, Fig. 4a).

There was a significant, negative effect of depth on the responses: in the models, the depth coefficient (β_depth_) was significant in all the cases explaining biomass [β_depth_ = −0.09; 95% CrI = −0.11, −0.08; P (β_depth_ < 0) = 1], secondary production [β_depth_ = −0.13; 95% CrI = −0.11, −0.08; P (β_depth_ < 0) = 1] and diversity [β_depth_ = −0.13; 95% CrI = −0.20, −0.06; P (β_depth_ < 0) = 1] of eumetazoan invertebrates, ciliates and flagellates (Fig. 4a). Furthermore, the reduction in biomass and secondary production with increasing depth varied significantly depending on the taxonomic group (Fig. 4a). Eumetazoa invertebrates showed the most abrupt reduction in biomass and secondary production with depth, followed by ciliates, and finally, flagellates. Eumetazoan invertebrates dominate biomass from the surface to 20 cm in depth, followed by ciliates and finally flagellates. Then, flagellates dominate total biomass below 20 cm where eumetazoan invertebrates are almost absent from the streambed and ciliate numbers are low (Fig. 4a).

The vertical hydrodynamic conditions were also an important predictor variable in our models, at least for biomass and secondary production. Biomass and secondary production were significantly higher in DW sites than in UW sites (Fig. 4a). In contrast, diversity values did not show any clear relationship with vertical hydrodynamic conditions (hydrodynamics as the non-significant coefficient in Poisson-GLMM). Therefore, reduction in Shannon-Wiener diversity with increasing depth displayed a similar behaviour, independently of the vertical exchange of water [model equations, fitted coefficients, 95% CrI and probability (P) that the coefficient is different from 0 (β ≠ 0) are available as Supplementary Material: Appendix 5 and Appendix 6)].

### Community structure

We were able to detect the depth at which the benthic assemblage is replaced by the hyporheic assemblage in our system. After ANOSIM analysis, significant differences between surface and sub-surface assemblages were detected between 5 and 10 cm layers under UW conditions (only 62% of similarity), and between 10 and 15 cm layers under DW conditions (Fig. 4b). Therefore, it was also possible to delineate the boundary between the BZ and the HZ purely based on biocenosis characteristics, and determine that the position of this boundary in the sediment differed depending on the direction of water flow. Our results showed that, at least for the limit between benthos and hyporheos (our main response), vertical differences in assemblage structure were significantly higher than horizontal differences (comparing between sites). We also found that the degree of change between the surface and deeper layers differed considerably depending on the vertical hydrodynamic conditions (the similarity analysis 1 – Bray-Curtis index; Fig. 4b). Under UW conditions, community structure varied dramatically with depth often scoring 0 below 30 cm depth (studied organisms were not found in this environment; Supplementary Material: Appendix 4 and Fig. S2). In contrast, under DW conditions, community structure was more consistent and organisms colonized deeper layers of the streambed (Fig. 4b). Regardless of the depth at which the boundary between both communities appeared, similarity of benthos and hyporheos was consistent between sites (Supplementary Material: Fig. S3). Although our study design did not allow inferential comparative tests between sampling dates, we observed that the reported differences between assemblages at the surface and in deeper layers persisted throughout the study period for both vertical hydrodynamic treatments (Supplementary Material: Appendix 7), suggesting that the location of the boundary between assemblages was stable, at least during the study period.

## Discussion

### *Hypothesis 1:*

*the reduction in diversity, biomass and secondary productivity with increasing depth in the streambed will depend on the body size of the taxonomic group*.

The decline of biomass, secondary production and diversity of streambed populations with increasing depth has been widely reported in the literature and our results support these findings^12,13,16,23,30,40,65,66^. However, no previous publications have quantified the relationship of these responses with depth in the streambed across a range of taxa with varying body sizes (eumetazoan invertebrates, ciliates and flagellates). Our study clearly showed that the decline of biomass and secondary production was dependent on the taxonomic group; taxa with smaller body sizes, lower metabolic rate and higher tolerance to low oxygen levels penetrated deeper into the sediment. Schmid-Araya^23^ also found that the abundance of flagellates increased in comparison to larger ciliates at deeper layers in the streambed. As previously suggested^17^, including the taxonomic group interaction term notably improved previous predictive models on depth–related biomass and secondary production of streambed assemblages and highlighted the necessity of including small fauna, such as ciliates and flagellates, when determining the total production of streambed systems^46^.

It is intuitive that the larger body-size of eumetazoan invertebrates constrains their ability to colonise agglomerated sediments^18,19,21^ and that the smaller size of ciliates and especially flagellates enables them to colonise the more compacted pore-space in deeper layers and dominate the assemblage in terms of biomass and secondary production. However, in our system there is also a strong redox gradient with depth which may additionally constrain eumetazoan invertebrates because they are highly dependent on oxygen availability in the streambed^19^. In contrast Protozoa include taxa known to support, and even prefer, anaerobic conditions^16,43^; unfortunately the level of protozoan taxonomic resolution in our study was insufficient to determine whether such groups were present in the community. The environmental filtering concept has been traditionally applied to determine community identity at large spatial scales (such as altitudinal and latitudinal gradients)^67^. Our findings suggest that micro-scale filters such as body size and metabolic requirements play a role in determining the community composition of streambed sediments and that environmental filtering is an important driver of gradients in the functional characteristics of organisms (i.e.^29,68^). However, other factors that we did not measure (e.g. food sources, predation, competition) may also influence the observed vertical gradients in the community^69,70^.

### *Hypothesis 2:*

*Community biomass, productivity and diversity will be significantly higher in DW flow conditions in comparison to UW flow conditions*.

Biomass and production also differed significantly depending on the vertical flow direction, mostly supporting our second hypothesis. In DW zones surface water ingress resulted in markedly lower redox potential at deeper depths than in UW zones. This promoted the establishment of assemblages that had greater biomass and production than those in UW zones. This idea has been hypothesised previously^71^, however, our interdisciplinary approach to determining vertical flow conditions and the resident community in the streambed sediments enabled us, for the first time, to confirm the link between streambed flow direction and productivity of streambed assemblages. We demonstrated the role of DW sites as significant hot spots of productivity, and therefore carbon processing in freshwater systems. We did not detect any effect of vertical hydrodynamic conditions on Shannon-Wiener diversity measurements, implying that there was a proportional simplification of the streambed assemblage through depth, independent of the vertical flux. Thus, similarly to Storey and Williams^64^, depth was the strongest predictor of the assemblage diversity models. Diversity is very low at all the study sites in the Erpe River (Supplementary Material, Appendix 2) and this may be why we did not detect any differences across vertical hydrodynamic conditions despite the many reports of their critical importance in the literature (i.e.^13,42,65,72^).

### *Hypotheses 3 & 4*

*: With increasing depth the benthic community will be replaced by a significantly different hyporheic community enabling the boundary between both communities to be delineated. Vertical hydrodynamic conditions will determine the depth at which this boundary occurs*.

Using a fine scale approach based on biocenosis features we detected the depth at which the hyporheos replaced the benthos, and showed that these two communities, composing the whole streambed assemblage, are measurable ecological entities with individual integrity. Thus our third hypothesis was supported. Our findings provide definitive quantitative evidence for previous suggestions that the benthos is replaced by the hyporheos with increasing depth into the sediment^65,70^. Furthermore, in support of our final hypothesis, the line of demarcation between benthos and hyporheos was governed by surface and groundwater influence; in DW sites, benthos colonized deeper layers because the benthic biotope extended deeper into the substratum and this finding appeared to be persistent over the study period (Supplementary Material: Appendix 6) although slight variations in the similarity values (1- Bray-Curtis index) suggest that location of the boundary between communities might vary marginally.

## Conclusions

Our study confirms that the HZ is a spatially ecotone between the surface stream and the deep groundwater^11^ and that the hyporheos is a discrete and measurable biological community. It also draws attention to the importance of including precise measurements of vertical flux direction when defining streambed system boundaries. Accurately defining natural system boundaries is an important aspect of designing ecological studies and for interpretation of results^40^, but it is also central to defining ecosystems^73^. Our study also supports the often mentioned necessity of multidisciplinary approaches in modern freshwater ecology. This strategy enabled us to assess the interplay of two central environmental filters (depth gradient and vertical hydrodynamic conditions) in driving the productivity, diversity and organization of streambed assemblages.

## Electronic supplementary material

Supplementary material

Supplementary Dataset 1

Supplementary Dataset 2

Supplementary Dataset 3

Supplementary Dataset 4

Supplementary Dataset 5
