# Author Correction: Environmental filtering and community delineation in the streambed ecotone

**DOI:** 10.1038/s41598-019-45692-0

**Published:** 2019-06-26

**Authors:** Ignacio Peralta-Maraver, Jason Galloway, Malte Posselt, Shai Arnon, Julia Reiss, Jörg Lewandowski, Anne L. Robertson

**Affiliations:** 1 Department of Life Sciences, Roehampton University, London, UK; 2 Leibniz-Institute of Freshwater Ecology and Inland Fisheries, Department Ecohydrology, Berlin, Germany; 3 Department of Environmental Science and Analytical Chemistry, Stockholm University, Stockholm, Sweden; 4 Zuckerberg Institute for Water Research, The Jacob Blaustein Institutes for Desert Research, Ben-Gurion University of the Negev, Midreshet Ben-Gurion, Israel; 5 Geography Department, Humboldt University, Berlin, Germany

Correction to: *Scientific Reports* 10.1038/s41598-018-34206-z, published online 26 October 2018

In the Supplementary Dataset 4 originally published with this Article, the data was incorrectly ordered. This error has been corrected in the Supplementary Dataset 4 that now accompanies the Article.

