# Supplementary material for: Environmental filtering and community delineation in the streambed ecotone
Source: Sci Rep. 2018 Oct 26;8:15871. doi: 10.1038/s41598-018-34206-z (PMC6203860; doi:10.1038/s41598-018-34206-z)

Supplementary material for:

Environmental filtering and community delineation in the streambed ecotone

Ignacio Peralta-Maraver, Jason Galloway, Malte Posselt, Shai Arnon, Julia Reiss, Jörg Lewandowski & Anne L. Robertson.

Index

Appendix 1 2

Appendix 2 3

Appendix 3 4

Appendix 4 5

Appendix 5 6

Appendix 6 7

Appendix 7 8

Fig S1 9

Fig S2 10

Fig S3 11

Appendix 1. Inputs parameters used in the one-dimensional advection-diffusion equations during VFLUX2 routines.

Parameter	Value	Description	Notes
- **rfactor**	12	- A positive integer factor by which to reduce the sampling rate.	- We used a reduced sampling rate of 12 samples per fundamental cycle is recommended in order to eliminate spurious filtration artifacts.
- **windows**	1	- A positive integer determining the number of sensor-spacings. VFLUX2 will calculate flux between all the sensor pairs that are separated by the window.	- We had 8 sensors in the profile and windows =1. VFLUX2 calculated fluxes between sensors 1-2, 2-3, 3-4, and 4-5, 5-6, 6-7, and 7-8.
- **Pf**	1	- Period of the fundamental temperature signal to filter and use for flux calculations (in days).	
- **n**	0.28	- Total porosity of the sediment.	- Default value for sandy sediments.
- **beta**	0.001	- Dispersity (m).	- Default value for sandy sediments.
- **Kcal**	0.0045	- Thermal conductivity, in cal/(s cm °C).	- Default value for sandy sediments.
- **Cscal**	0.5	- Volumetric heat capacity of the sediment.	- Default value for sandy sediments.
- Cwcal	1.0	- Volumetric heat capacity of the wáter.	- Default value for sandy sediments.

Appendix 2: Identified taxa list including colonization depth (in cm) in downwelling (DW) sites and upwelling (UW) sites.

**Taxa**	**Group**	**Depth DW-Sites**							**Depth UW-Sites**						
		**5**	**10**	**15**	**20**	**25**	**30**	**35**	**5**	**10**	**15**	**20**	**25**	**30**	**35**
Asellidae	Invertebrate	×							x						
Baetis sp.	Invertebrate	×													
Bdelloidea	Invertebrate	×	×	×					×	×					
Ceratopogonidae	Invertebrate	×	×						×						
Chironomidae (pupae)	Invertebrate	×							×						
Chironomini	Invertebrate	×	×		×	×			×	×	×				
Cladocera	Invertebrate	×	×						×	×					
Gammarus sp.	Invertebrate	×							×						
Harpacticoida	Invertebrate	×	×	×					×	×	×				
Hexatomini	Invertebrate	×													
Hirudinea	Invertebrate	×	×	×					×						
Hydra	Invertebrate	×	×						×						
Nauplii	Invertebrate		×						×						
Nematoda	Invertebrate	×	×	×	×				×	×	×	×			
Oligochaeta	Invertebrate	×	×	×	×	×			×	×	×				
Oligochaeta (eggs)	Invertebrate	×	×						×						
Orthocladinae	Invertebrate	×	×						×		×				
Ostracoda	Invertebrate	×	×	×	×				×						
Simulinii	Invertebrate	×							×						
Tanypodinae	Invertebrate	×	×						×	×	×				
Tanytarsini	Invertebrate	×	×	×	×				×	×	×				
Tardigrade	Invertebrate	×													
Cyrtophorida	Ciliate	×	×						×	×	×	×			
Gymnostomatida	Ciliate	×	×	×	×				×	×		×			
Heterotrichida	Ciliate	×	×	×	×	×			×	×	×	×			
Hymenostomata	Ciliate	×	×	×	×	×			×	×	×	×			
Hypotrichia	Ciliate	×	×	×	×	×			×	×	×	×			
Monogonta	Ciliate	×	×	×					×						
Peritrichia	Ciliate	×	×						×	×					
Pleurostomatida	Ciliate	×	×	×					×	×	×				
Prostomatida	Ciliate	×	×												
Suctoria	Ciliate	×	×						×						
Flagellate	Flagellate	×	×	×	×	×	×	×	×	×	×	×	×	×	

Appendix 3: Frequency of body sizes (log_{10} biovolume, nL) in the streambed assemblage of the Erpe River. Dots represent mean values for flagellates (red), ciliates (blue and eumetazoa invertebrates (invertebrates, orange). Horizontal lines represent the standard deviations. It was possible to discriminate three size groups, which clearly corresponded with the studied taxonomic groups.

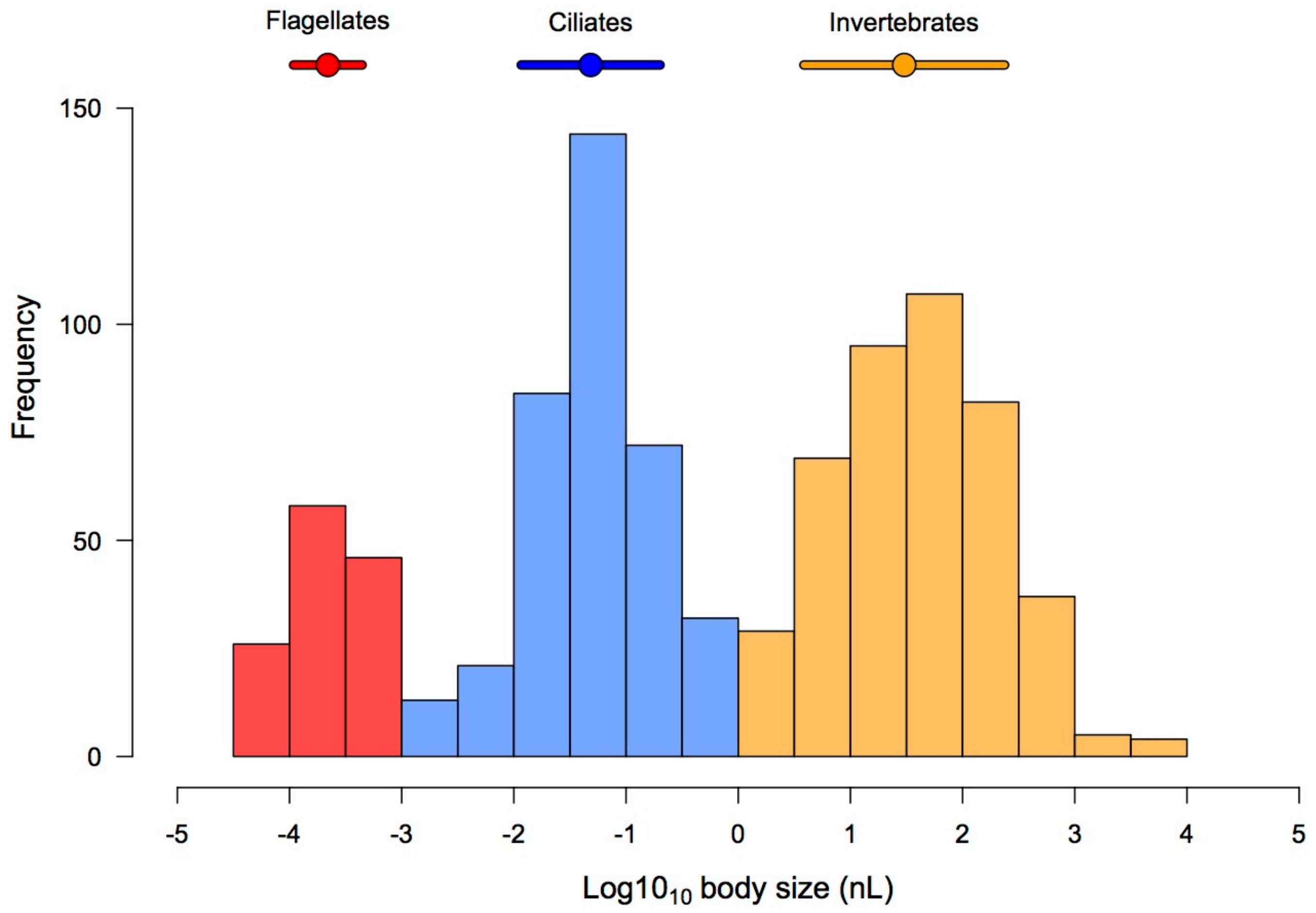

Appendix 4: Mean and standard deviation (SD) values of biomass (mg C/L), production (mg C/L month) and Shannon-Wiener diversity index (Diversity) of different taxonomic groups, at upwelling sites (UW-sites) and down welling sites (DW), and at different depths.

Group	Depth	DW Sites		UW Sites	
		P (SD)	B (SD)	P (SD)	B (SD)
Invertebrates	5	37.844 (85.219)	103.350 (359.098)	20.052 (47.794)	52.720 (169.902)
	10	11.566 (52.662)	23.942 (124.416)	0.650 (1.324)	3.547 (17.787)
	15	5.760 (24.727)	2.197 (7.429)	0.091 (0.139)	0.230 (0.271)
	20	0.019 (0.016)	0.167 (0.280)	0.009 (NA)	0.046 (0.016)
	25	0.140 (0.195)	0.281 (0.141)	0.000	0.000
	30	0.000	0.000	0.000	0.000
	35	0.000	0.000	0.000	0.000
Ciliates	5	1.363 (1.049)	0.160 (0.270)	1.506 (2.139)	0.233 (0.355)
	10	0.282 (2.044)	0.130 (0.465)	0.515 (0.632)	0.073 (0.086)
	15	0.290 (0.347)	0.036 (0.057)	0.096 (0.079)	0.021 (0.036)
	20	0.064 (0.099)	0.008 (0.012)	0.015 (0.015)	0.004 (0.006)
	25	0.004 (0.002)	0.001 (0.001)	0.000	0.000
	30	0.000	0.000	0.000	0.000
	35	0.000	0.000	0.000	0.000
Flagellates	5	1.44 (0.356)	0.028 (0.006)	1.638 (0.525)	0.031 (0.015)
	10	0.968 (0.356)	0.020 (0.012)	0.651 (0.279)	0.015 (0.010)
	15	0.803 (0.068)	0.016 (0.010)	0.483 (0.225)	0.010 (0.008)
	20	0.438 (0.088)	0.009 (0.006)	0.238 (0.200)	0.006 (0.006)
	25	0.397 (0.051)	0.010 (0.007)	0.156 (0.067)	0.006 (0.006)
	30	0.297 (0.237)	0.007 (0.004)	0.065 (0.019)	0.007 (0.004)
	35	0.144 (0.051)	0.008 (0.008)	0.000	0.000
Diversity	5	0.360 (0.107)		0.310 (0.116)	
	10	0.240 (0.063)		0.325 (0.068)	
	15	0.140 (0.073)		0.137 (0.082)	
	20	0.100 (0.068)		0.057 (0.062)	
	25	0.023 (0.031)		0.000	
	30	0.000		0.000	
	35	0.000		0.000	

Appendix 5. Predictive models equations. (**1**) Linear Mixed Model in which responses $\hat{Y}$ (*Biomass* or *Production*) depend on the fixed intercept (β_0), random intercept (a_{site}), $Depth_i$ (continuous variables), $Group_j$ (factor with 3 levels: Invertebrates, ciliates and flagellates), interaction between $Depth_i$ and $Group_j$, and $Hydrodynamics_z$ (factor with 2 levels: UW and DW conditions). Both fixed residuals (ε_{ijz}) and random intercept follow a normal distribution. (**2**) Poisson Generalised Linear Mixed Model in which response (predicted mean of Shannon-Wiener diversity, η_{iz}) depend on the fixed intercept (β_0), random intercept (a_{site}), $Depth_i$ (continuous variables) and $Hydrodynamics_z$ (factor with 2 levels: UW and DW conditions).

1) $\mathbf{Log_{10}}(\hat{Y}_{ijz}) = \boldsymbol{\beta_0} + \mathbf{a}_{\text{site}} + \boldsymbol{\beta_1} \times Depth_i : \boldsymbol{\beta_2} \times Group_j + \boldsymbol{\beta_3} \times Hydrodynamics_z + \boldsymbol{\varepsilon_{ijz}}$

$\hat{Y}_{ijz} \sim \text{Norm}(0, \boldsymbol{\sigma^2_d})$

$\varepsilon_{ijz} \sim \text{Norm}(0, \boldsymbol{\sigma^2})$

$\boldsymbol{a_{site}} \sim \text{Norm}(0, \boldsymbol{\sigma^2_{site}})$

2) $\boldsymbol{\eta_{iz}} = \boldsymbol{\beta_0} + \mathbf{a}_{\text{site}} + \boldsymbol{\beta_1} \times Depth_i + \boldsymbol{\beta_2} \times Hydrodynamics_z + \boldsymbol{\varepsilon_{ijz}}$

$Shannon_{iz} \sim \text{Pois}(\mu_{iz})$

$\text{E}(Shannon_{iz}) = \mu_{iz}$

$\mu_{iz} = \eta_{iz}$

$\boldsymbol{\varepsilon_{ijz}} \sim \text{Norm}(0, \boldsymbol{\sigma^2_d})$

$\boldsymbol{a_{site}} \sim \text{Norm}(0, \boldsymbol{\sigma^2_{site}})$

Appendix 6. Fitting models-coefficients and probability estimates. Mean, standard errors (SE), lower and upper credible intervals (2.5 and 97.5% CrI) and probability that the coefficient is different from 0 [P(β≠0)].

Log_{10}(*Biomass*)	Mean	SE	2.5% CrI	97.5% CrI	P(β≠0)
Intercept	-0.523	0.117	-0.757	-0.293	1.000 *
Depth	-0.094	0.009	-0.113	-0.076	1.000 *
Group (flagellate)	-0.911	0.206	-1.321	-0.511	1.000 *
Group (invertebrate)	2.126	0.143	1.854	2.416	1.000 *
Hydrodynamics (UW)	-0.159	0.074	-0.295	-0.009	0.983 *
Depth x Group (flagellate)	0.064	0.013	0.038	0.090	1.000 *
Depth x Group (Invertebrate)	-0.078	0.014	-0.106	-0.052	1.000 *
**Log_{10}(*Production*)**					
Intercept	0.516	0.190	0.136	0.894	0.997 *
Depth	-0.105	0.014	-0.133	-0.076	1.000 *
Group (flagellate)	0.030	0.398	-0.752	0.794	0.530
Group (invertebrate)	0.898	0.231	0.459	1.349	1.000 *
Hydrodynamics (UW)	-0.387	0.102	-0.587	-0.193	1.000 *
Depth x Group (flagellate)	0.063	0.023	0.018	0.106	0.996 *
Depth x Group (Invertebrate)	-0.065	0.020	-0.104	-0.025	1.000 *
**Log_{10}(*Shannon*)**					
Intercept	-0.221	0.743	-1.662	1.289	0.609
Depth	-0.135	0.033	-0.199	-0.067	1.000 *
Hydrodynamics (UW)	-0.049	0.927	-1.811	1.821	0.561

Appendix 7. Bray-Curtis similarity matrix of the assemblage structure between depth layers in upwelling sites (UW) and downwelling sites (DW) every 7 days. Each matrix contains the numeric Bray-Curtis similarity value that resulted from comparing assemblage structure (composition and abundance) between sections every 7 days. Similarity values range from maximum similarity (1.00) to maximum dissimilarity (0.00).

		**UW Sites**							**DW Sites**						
		5cm	10cm	15cm	20cm	25cm	30cm	35cm	5cm	10cm	15cm	20cm	25cm	30cm	35cm
**week 1**	5cm	1,00							1,00						
	10cm	0,79	1,00						0,87	1,00					
	15cm	0,48	0,65	1,00					0,85	0,96	1,00				
	20cm	0,33	0,46	0,77	1,00				0,58	0,68	0,71	1,00			
	25cm	0,24	0,35	0,61	0,82	1,00			0,41	0,48	0,51	0,76	1,00		
	30cm	0,17	0,25	0,45	0,64	0,80	1,00		0,22	0,27	0,28	0,46	0,66	1,00	
	35cm	0,00	0,00	0,00	0,00	0,00	0,00	1,00	0,22	0,27	0,28	0,46	0,66	1,00	1,00
**week 2**	5cm	1,00							1,00						
	10cm	0,58	1,00						0,86	1,00					
	15cm	0,31	0,61	1,00					0,65	0,76	1,00				
	20cm	0,29	0,57	0,94	1,00				0,43	0,52	0,73	1,00			
	25cm	0,23	0,47	0,82	0,87	1,00			0,41	0,49	0,70	0,97	1,00		
	30cm	0,00	0,00	0,00	0,00	0,01	1,00		0,29	0,35	0,52	0,76	0,79	1,00	
	35cm	0,00	0,00	0,00	0,00	0,01	0,92	1,00	0,00	0,00	0,00	0,00	0,00	0,01	1,00
**week 3**	5cm	1,00							1,00						
	10cm	0,55	1,00						0,81	1,00					
	15cm	0,39	0,79	1,00					0,70	0,88	1,00				
	20cm	0,26	0,56	0,74	1,00				0,42	0,56	0,66	1,00			
	25cm	0,00	0,00	0,00	0,01	1,00			0,26	0,36	0,44	0,72	1,00		
	30cm	0,00	0,00	0,00	0,01	0,91	1,00		0,18	0,26	0,31	0,54	0,80	1,00	
	35cm	0,00	0,00	0,00	0,01	0,83	0,92	1,00	0,00	0,00	0,00	0,00	0,01	0,02	1,00
**week 4**	5cm	1,00							1,00						
	10cm	0,80	1,00						0,89	1,00					
	15cm	0,31	0,43	1,00					0,60	0,68	1,00				
	20cm	0,11	0,15	0,38	1,00				0,57	0,65	0,96	1,00			
	25cm	0,00	0,00	0,00	0,00	1,00			0,44	0,51	0,80	0,84	1,00		
	30cm	0,00	0,00	0,00	0,00	1,00	1,00		0,35	0,41	0,67	0,70	0,86	1,00	
	35cm	0,00	0,00	0,00	0,00	1,00	1,00	1,00	0,19	0,23	0,40	0,42	0,54	0,67	1,00

Fig S1. Scheme of the sampling design during time (from week 1 to week 4): Arial view of the 1 m^2 area (grey square) in which corers (C1, C2 and C3) were extracted and sampling devices [Water level data loggers (Diver), thermal lance (T.Lance) and Peeper] installed. Note that locations of the sampled cores were alternated weekly to avoid disrupting effects on community assemblage.

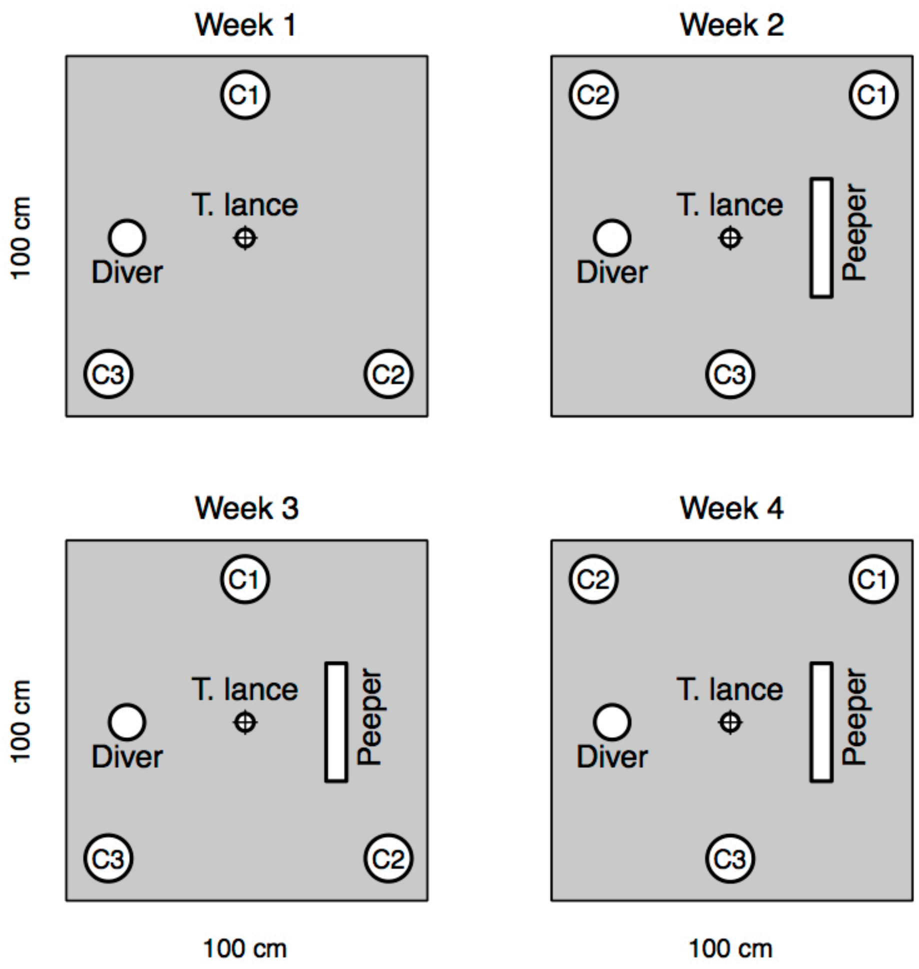

Fig S2. Abundance (Log_{10}) of different groups through depth profile (individuals/ L) in upwelling (UW) sites and downwelling sites (DW).

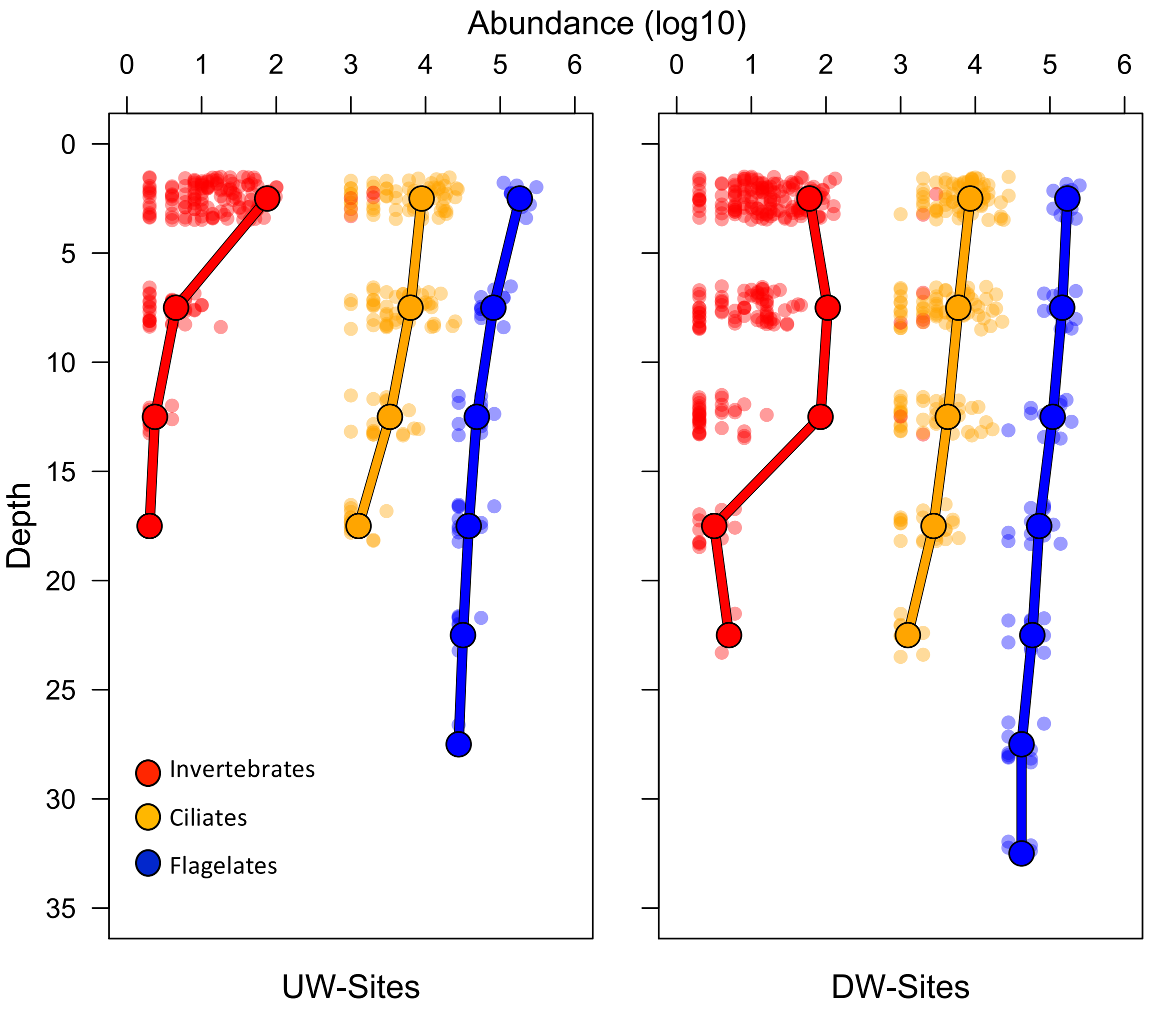

Fig S3. Clustering of the streambed assemblage a different depth layers under downwelling (DW in blue) and upwelling (conditions).

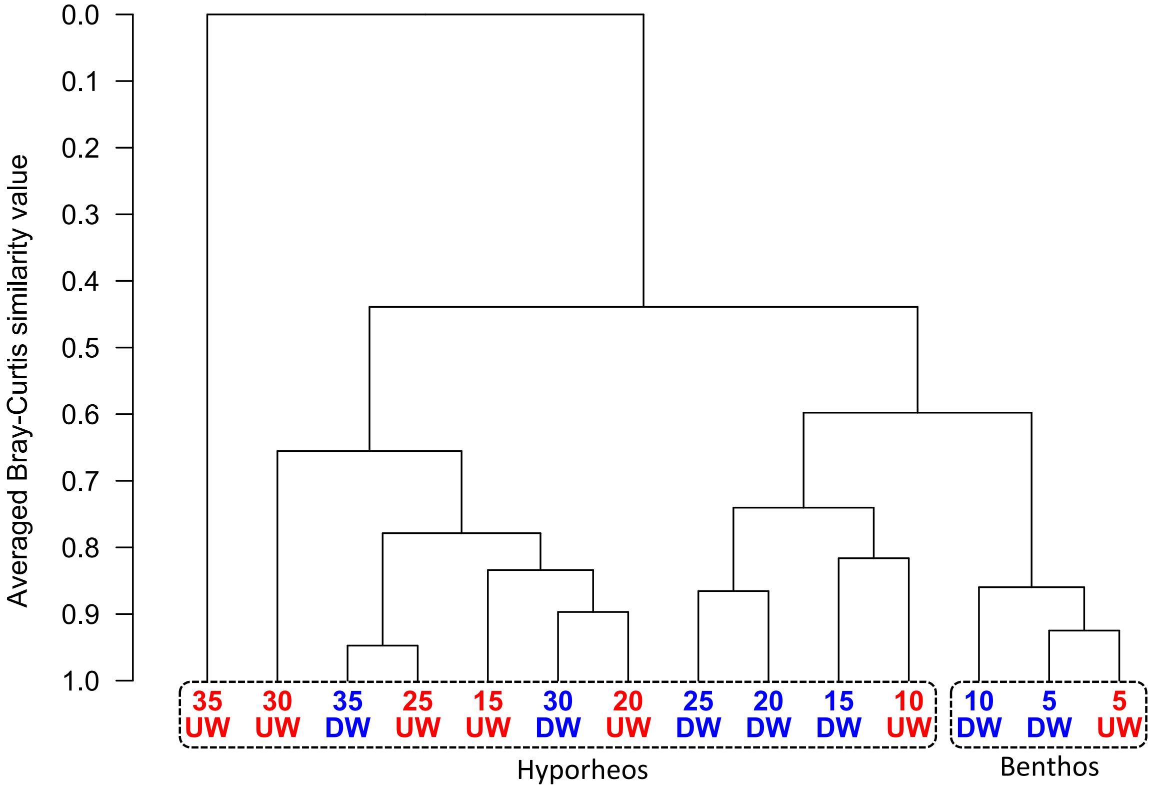

Supplement: Supplementary file 1 — Supplementary material [file 41598_2018_34206_MOESM1_ESM.pdf]
